# Supplementary material for: Coincident onset of charge order and pseudogap in a homogeneous high-temperature superconductor
Source: Nat Commun. 2025 Apr 15;16:3579. doi: 10.1038/s41467-025-58870-8 (PMC12000310; doi:10.1038/s41467-025-58870-8)
Supplement: Supplementary file 1 — Supplementary Information [file 41467_2025_58870_MOESM1_ESM.pdf]

Supplementary information for Coincident onset of charge order and pseudogap in a homogeneous high-temperature superconductor

D. Betto,[1,*] S. Nakata,[2,*] F. Pisani,[1] Y. Liu,[2] S. Hameed,[2] M. Knauft,[2] C. T. Lin,[2] R. Sant,[1] K. Kummer,[1] F. Yakhou,[1] N. B. Brookes,[1] M. Le Tacon,[3] B. Keimer,[2] and M. Minola[2,†]

[1]*European Synchrotron Radiation Facility (ESRF), BP 220, F-38043 Grenoble Cedex, France*

[2]*Max Planck Institute for Solid State Research, Heisenbergstraße 1, D-70569 Stuttgart, Germany*

[3]*Institute for Quantum Materials and Technologies, Karlsruhe Institute of Technology, Kaiserstr. 12, 76131 Karlsruhe, Germany*

[*] These two authors contributed equally.

[†] Corresponding author, email address: m.minola@fkf.mpg.de

SUPPLEMENTARY NOTES

Supplementary Note 1. Magnetometry measurement

Supplementary Figure 1 displays the magnetization curve of the $YBa_2Cu_4O_8$ (Y124) sample used in the present RIXS study. T_c ($\sim$ 81.1 K) was defined as the middle point of the diamagnetic response.

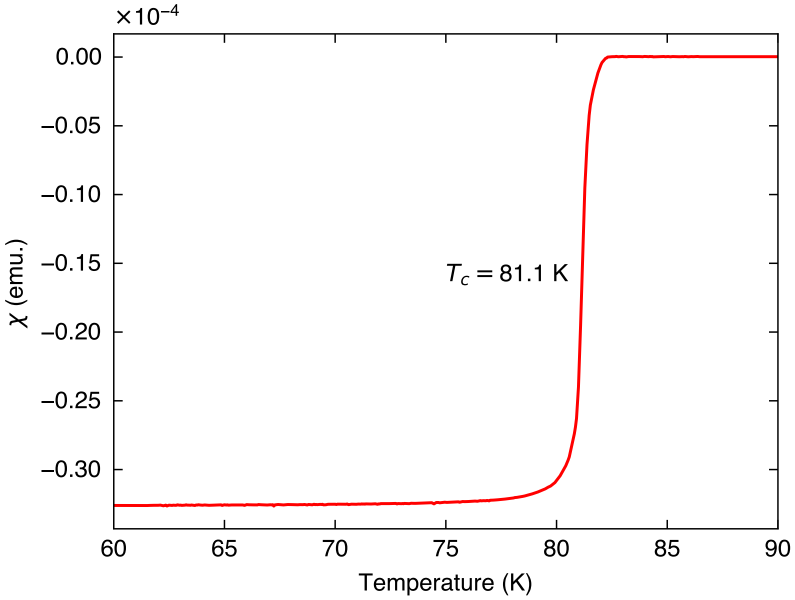

Supplementary Figure 1. **Magnetization curve of the Y124 sample investigated in the present RIXS study.**

Supplementary Note 2. Twin domains

Supplementary Figure 2 shows the surfaces of the sample studied in the present work. The sample thickness ($\sim$ 50 μm) is well below the in-plane dimensions. These images were obtained with a polarized microscope. The brightness of the images depends on the relative angle between the polarizer and the in-plane crystal axes. The domain with the a-axis along the vertical (horizontal) direction provides bright (dark) contrast in the present setup. Most of the region on both surfaces is characterized by a single color, indicating a large untwinned domain that was naturally obtained although the sample was not mechanically detwinned. Moreover, the in-plane crystal axes of the largest domain on the top and bottom surfaces are parallel to each other. Therefore the sample is likely to be nearly untwinned in a large fraction of the sample volume. In the RIXS experiments, the surface 1 was selected to be exposed to photons.

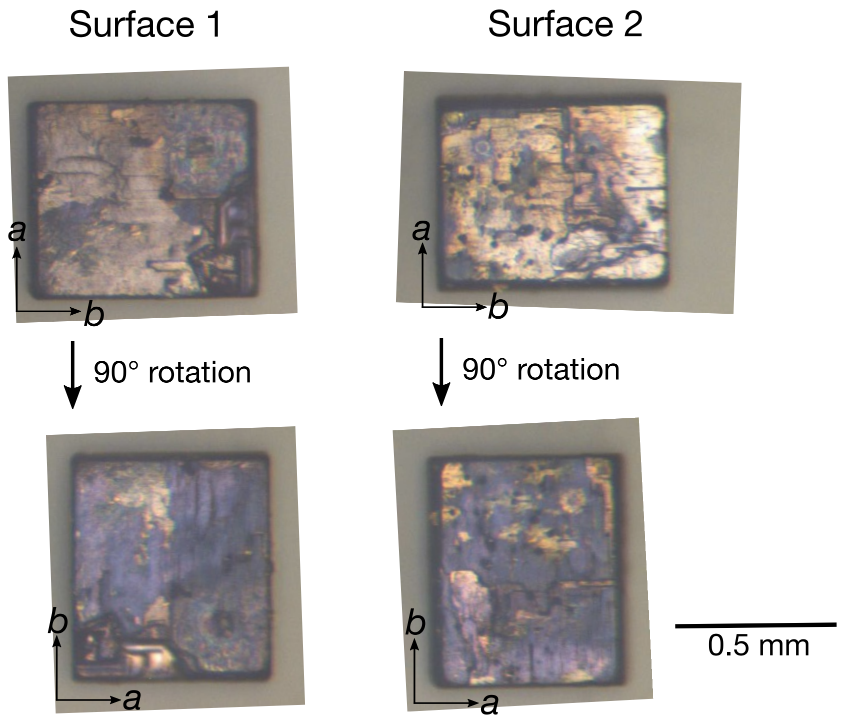

Supplementary Figure 2. **Sample surfaces viewed through the polarized microscope.** Both top (surface 1) and bottom (surface 2) surfaces are shown. The azimuth angle of the sample is rotated by 90 degrees between the upper and lower images.

Supplementary Note 3. Quasielastic intensities at various temperatures

Supplementary Figures 3 and 4 show quasielastic intensities corresponding to charge order propagating along the b-axis taken at various temperatures, together with the results of fits to Lorentzian profiles on a linear background. Figure 3b in the main text shows the amplitude of the Lorentzians resulting from these fits.

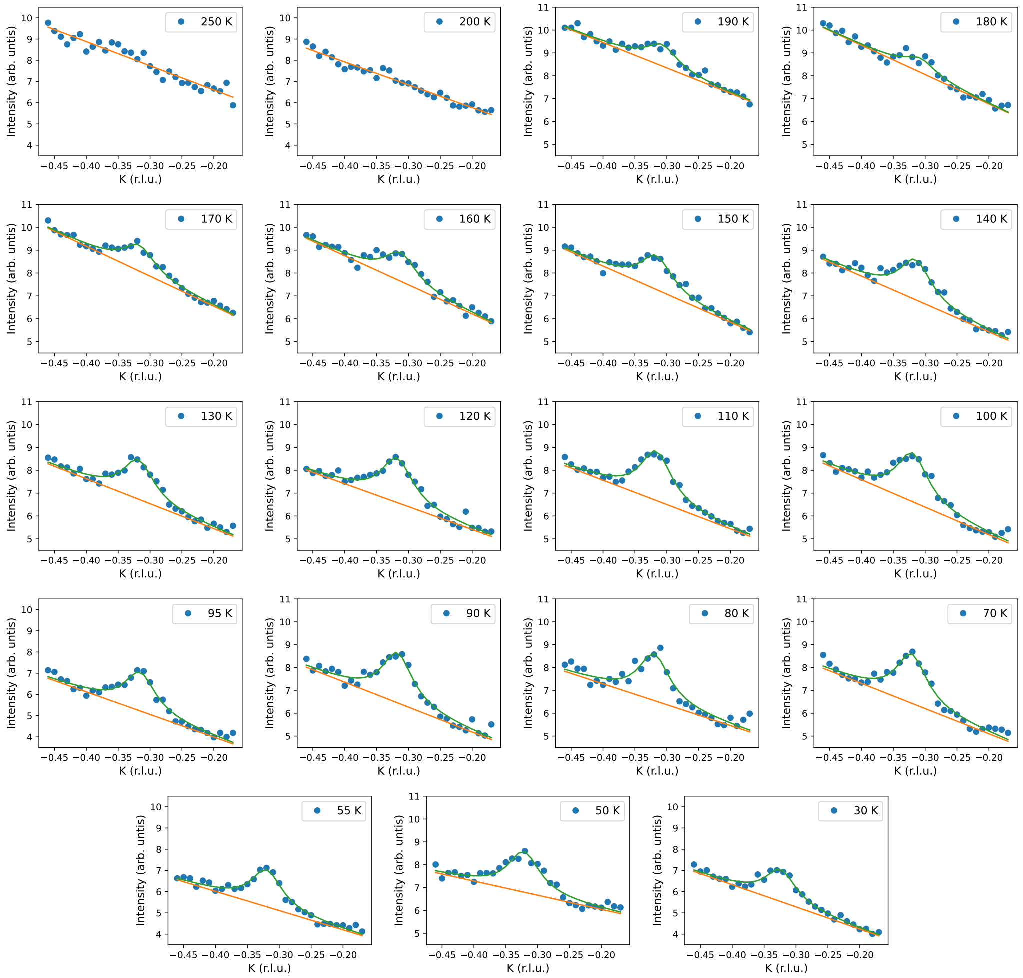

Supplementary Figure 3. **Quasielastic intensity.** The RIXS spectra between ± 100 meV of the elastic energy were integrated. The green and orange curves represent the Lorentzian fit and linear background, respectively.

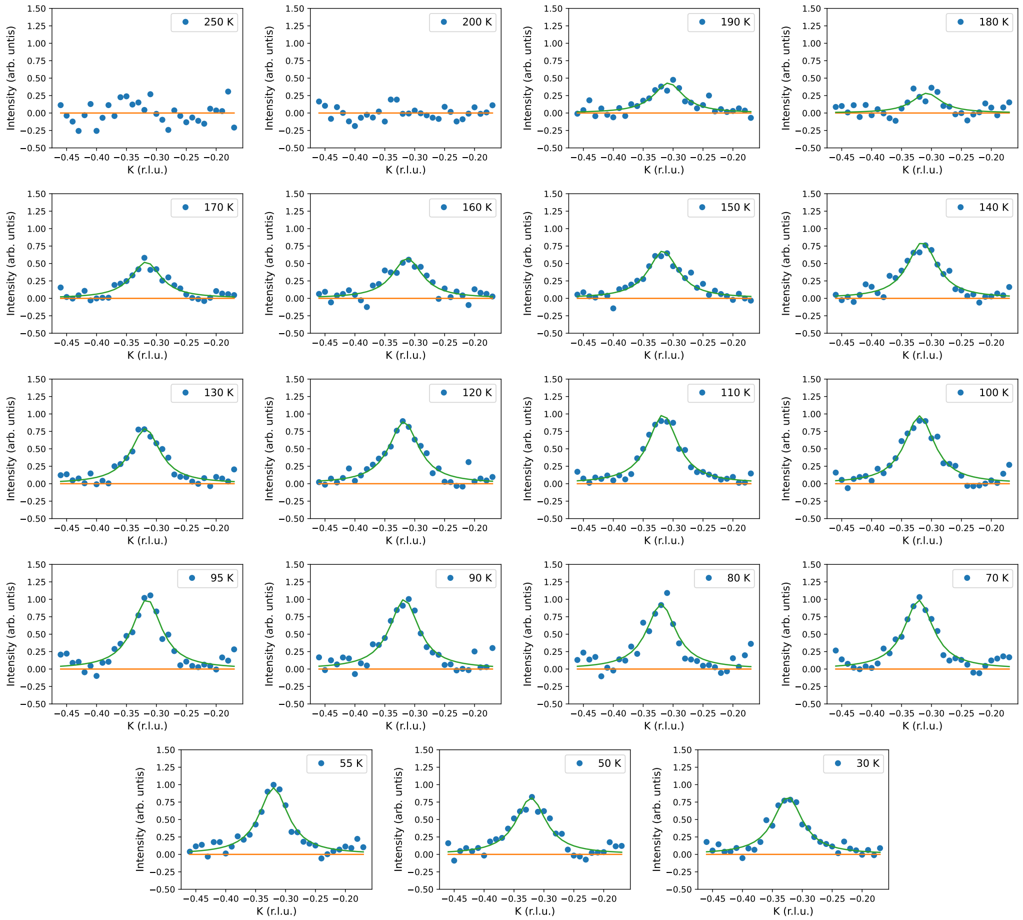

Supplementary Figure 4. **Quasielastic peak.** The RIXS spectra between ± 100 meV of the elastic energy are integrated. The linear background was subtracted. The green curves represent results of the Lorentzian fits. The peak intensity at all temperatures was normalized to the one at the temperature which gives the maximum peak intensity.

Supplementary Note 4. Transverse scan

Supplementary Figure 5 shows longitudinal (blue) and transverse (red) scans across the incommensurate superstructure reflection at (0, 0.32). The orange ellipse indicates that the transverse width is about a factor of three larger, reflecting an elongated shape of the charge-ordered domains, as previously observed in Y123 [1, 2].

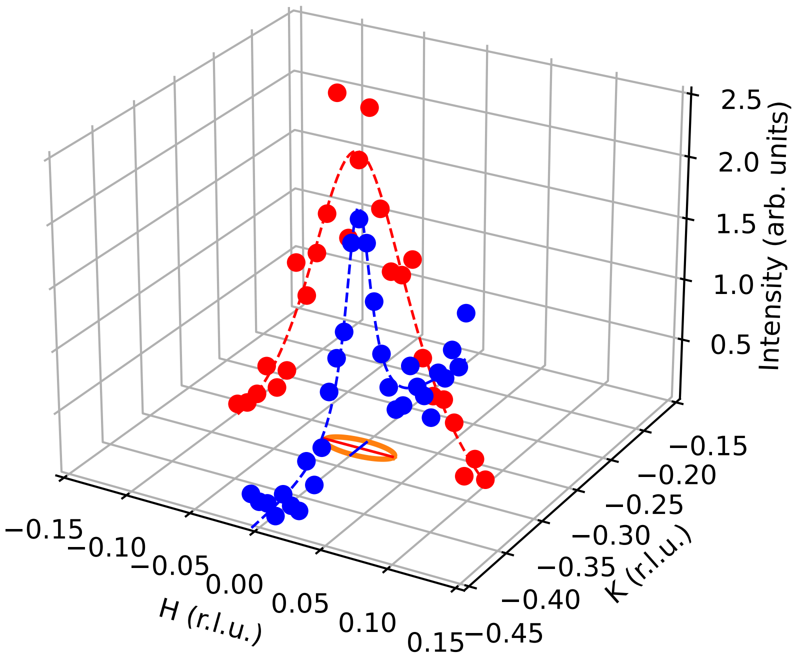

Supplementary Figure 5. **Longitudinal (blue) and transverse (red) scans across (0, 0.32).**

Supplementary Note 5. Peak width of quasielastic peaks

We note the temperature-independent width of the quasielastic peaks shown in Fig. 3a. Using a Lorentzian and linear background, we first unbiasedly fitted the data with variable peak intensity and width at each temperature. At this stage, it was found that the FWHMs showed no specific trend in temperature (Inset of Fig. 3b and Supplementary Fig. 6).

We used a mean value of the FWHMs obtained in the first fits as a constant fixed width for another peak fits. The peak intensity obtained in these fits is shown in Fig. 3b of the main text.

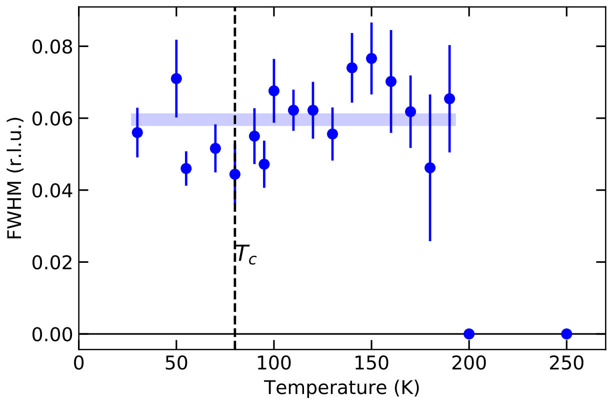

Supplementary Figure 6. **Temperature evolution of the FWHM of the quasielastic peaks.** Error bars are standard deviations of the fits. The blue horizontal line is a mean value of the FWHMs obtained in the original fits.

Supplementary Note 6. Temperature dependence of the CDW intensity in Y123

Supplementary Figure 7 shows the temperature evolution of the CDW for Y123 bulk crystals and thin films measured by RIXS (identical to the technique employed in the present work), REXS, non-resonant inelastic x-rays scattering (NRIXS), and hard x-ray diffraction (NREXS). The temperature dependence of the CDW intensity from the bulk crystals are nearly identical regardless of the experimental methods. In particular, energy-resolved RIXS and energy-integrated REXS data on Y123 bulk crystals are indistinguishable within the experimental error [3]. It is thus appropriate to compare the REXS data of Y123 and RIXS data of Y124 in Fig. 3b in the main text.

For completeness, Supplementary Figure 7 also reproduces REXS data from a study of thin-film samples of underdoped Y123 on substrates with different levels of epitaxial strain [4]. This study reported a parallel trend of the strain dependence of T_{CDW} extracted from REXS and T^* determined by resistivity measurements, in qualitative agreement with our results on Y124. In contrast to Y124 crystals, however, NMR, NQR, IR, and Raman measurements were not reported for the Y123 thin-film samples, and T^* was determined based on an assessment of the deviation of the dc resistivity from T-linear behavior. Note that this procedure has been debated in the literature [5]. The T-dependent CDW order is shown for one film with 10 nm thickness (gray dots in Supplementary Fig. 7, right panel), and for this sample, the temperature range with T-linear resistivity extends only from $\sim$230 to 300 K (Fig. 3 in Ref. [4]). We also note that the charge redistribution at the surface and substrate interface of thin films adds another source of inhomogeneity. In particular, a four-unit-cell ($\sim$4.5 nm) thick buffer layer with reduced doping level was recently found in $DyBa_2Cu_3O_{7-\delta}$ thin films and attributed to missing CuO chains at the substrate interface [6]. The results reported for Y123 films reported in Ref. [4] are thus complementary to our study of Y124 bulk crystals, and call for further investigations of the effects of controlled disorder and inhomogeneity on the CDW and the pseudogap.

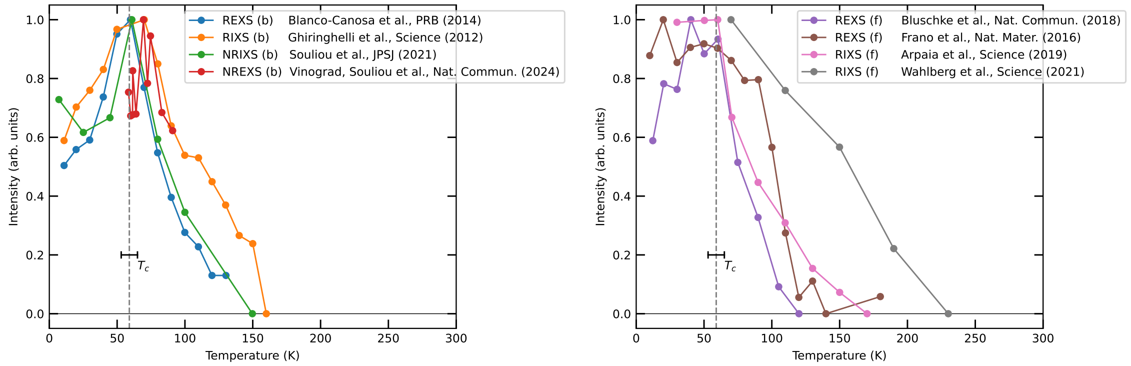

Supplementary Figure 7. **Temperature evolution of the CDW intensity in Y123.** The data were collected for Y123 bulk crystals (left) and thin films (right) by means of various types of x-ray scattering measurements (RIXS, REXS, NRIXS, NREXS) in prior studies [3, 4, 7–12].

Supplementary Note 7. Momentum space covered in Cu-L edge RIXS

Supplementary Figure 8 shows the momentum space accessible in Cu-L edge RIXS for Y124 and Y123. Since the c-axis length of Y124 is 27.25 Å, i.e. a bit more than double of that in Y123 (11.75 Å), the accessible momentum along the L direction is quite different between these two materials.

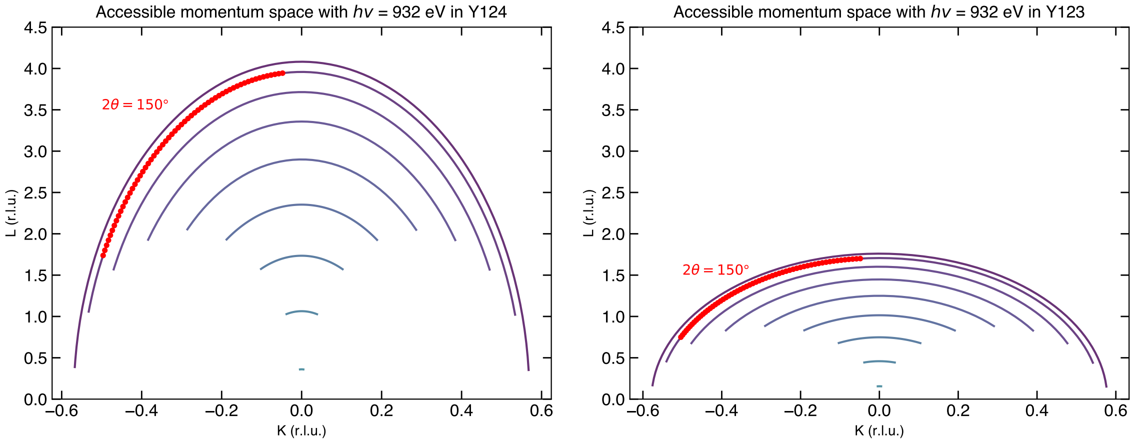

Supplementary Figure 8. **Accessible momentum space with the Cu-L edge absorption edge energy.** (Left) Y124. (right) Y123. The dark curves correspond with continuous theta scans from $\theta = 0$ to $\theta = 2\theta$ for each 2θ (= 10°, 30°, ..., 150°, 170°). The red curve represents the one with the highest reachable momentum at the Cu-L_3 edge with the ERIXS spectrometer and also the geometry used to collect the theta scans in Figs. 2a,b in the main text.

Supplementary Note 8. Chemical analyses

To ensure the quality of the sample used in the present RIXS study, we investigated composition ratio and possible contamination from the Y124 sample.

We first examined the composition ratio of the Y124 sample with energy dispersive x-ray spectroscopy (EDX). Based on the intensity of the EDX spectra, the atomic ratio of Y, Ba, Cu, and O of the sample was obtained (Supplementary Table 1). The composition ratio Y : Ba : Cu is estimated to be 0.98 : 2.08 : 3.94, which is in the proximity of the nominal composition ratio (1 : 2 : 4).

Measurement position	O-K	Cu-K	Y-L	Ba-L
1	49.95	28.23	6.97	14.85
2	47.16	30.05	6.83	15.96
3	51.29	27.33	7.01	14.37
4	51.19	27.2	7.07	14.54
5	50.6	27.51	7.12	14.77
6	46.53	30.58	7.17	15.73
7	49.69	28.23	7.05	15.02
8	51.46	27.21	6.98	14.36
9	49.72	28.22	7.11	14.94

Supplementary Table 1. Atomic ratio of the Y124 sample measured in the present RIXS experiment based on EDX measurements. The EDX measurements were repeated at nine different positions of the sample.

Moreover, we examined impurities using inductively coupled plasma optical emission spectrometer (ICP-OES). Since the sample was grown in an AlO_2 crucible using KOH flux, Al and K were tested as possible contamination. Ca was also checked because Ca was previously used in the furnace in which the Y124 sample was grown. The result of the ICP-OES measurements is summarized in Supplementary Table 2. All three elements are found to be less than ∼0.2 % of the total weight of the sample.

Sample	Sample weight (mg)	Al (weight %)	Ca (weight %)	K (weight %)
1	12.9215	0.092	0.010	0.199
2	16.8979	0.064	0.011	0.252
3	20.3868	0.072	0.007	0.189
Average	-	0.076 ± 0.014	0.009 ± 0.002	0.213 ± 0.034

Supplementary Table 2. Weight ratio of possible contamination in the Y124 sample measured with ICP-OES.

[1] Comin, R. *et al.* Broken translational and rotational symmetry via charge stripe order in underdoped $YBa_2Cu_3O_{6+y}$. *Science* **347**, 1335–1339 (2015).

[2] Kim, H.-H. *et al.* Charge Density Waves in $YBa_2Cu_3O_{6.67}$ Probed by Resonant X-Ray Scattering under Uniaxial Compression. *Phys. Rev. Lett.* **126**, 037002 (2021).

[3] Ghiringhelli, G. *et al.* Long-Range Incommensurate Charge Fluctuations in $(Y,Nd)Ba_2Cu_3O_{6+x}$. *Science* **337**, 821–825 (2012).

[4] Wahlberg, E. *et al.* Restored strange metal phase through suppression of charge density waves in underdoped $YBa_2Cu_3O_{7-\delta}$. *Science* **373**, 1506–1510 (2021).

[5] Hussey, N. E., Nozawa, K., Takagi, H., Adachi, S. & Tanabe, K. Anisotropic resistivity of $YBa_2Cu_4O_8$: Incoherent-to-metallic crossover in the out-of-plane transport. *Phys. Rev. B* **56**, R11423–R11426 (1997).

[6] Dawson, R. D. *et al.* Approaching Two-Dimensional Superconductivity in Ultrathin $DyBa_2Cu_3O_{7-\delta}$. *Phys. Rev. Lett.* **125**, 237001 (2020).

[7] Blanco-Canosa, S. *et al.* Resonant x-ray scattering study of charge-density wave correlations in $YBa_2Cu_3O_{6+x}$. *Phys. Rev. B* **90**, 054513 (2014).

[8] Souliou, S.-M. *et al.* In-plane Isotropy of the Low Energy Phonon Anomalies in $YBa_2Cu_3O_{6+x}$. *J. Phys. Soc. Jpn.* **90**, 111006 (2021).

[9] Vinograd, I. *et al.* Using strain to uncover the interplay between two- and three-dimensional charge density waves in high-temperature superconducting $YBa_2Cu_3O_y$. *Nat. Commun.* **15**, 3277 (2024).

[10] Bluschke, M. *et al.* Stabilization of three-dimensional charge order in $YBa_2Cu_3O_{6+x}$ via epitaxial growth. *Nat. Commun.* **9**, 2978 (2018).

[11] Frano, A. *et al.* Long-range charge-density-wave proximity effect at cuprate/manganate interfaces. *Nat. Mater.* **15**, 831 EP – (2016).

[12] Arpaia, R. *et al.* Dynamical charge density fluctuations pervading the phase diagram of a Cu-based high-T_c superconductor. *Science* **365**, 906–910 (2019).
